# Aging increases the susceptibility of cisplatin-induced nephrotoxicity

**DOI:** 10.1007/s11357-015-9844-3

**Published:** 2015-11-03

**Authors:** Jiagen Wen, Meizi Zeng, Yan Shu, Dong Guo, Yi Sun, Zhen Guo, Youhong Wang, Zhaoqian Liu, Honghao Zhou, Wei Zhang

**Affiliations:** 1 Department of Clinical Pharmacology, Xiangya Hospital, Central South University, Changsha, China; 2 Hunan Key Laboratory of Pharmacogenetics, Changsha, China; 3 Department of Pharmaceutical Sciences, School of Pharmacy, University of Maryland, Baltimore, MD USA; 4 Department of Pathology, The Second Xiangya Hospital of Central South University, Changsha, China

**Keywords:** Cisplatin, eGFR, Inflammatory response, Nephrotoxicity, Nrf2, Transporters

## Abstract

Cisplatin (CDDP) nephrotoxicity is one of the most common side effects in cancer treatment, causing the disruption of chemotherapy. In this study, we analyzed the influence of nongenetic factors on CDDP-induced nephrotoxiciy using the data from 182 CDDP-treated and 52 carboplatin (CBP)-treated patients. The mean change of eGFR (100 % to baseline) in CDDP-treated patients was −9.2 %, which was significantly lower than that in the population with CBP therapy. By using the chi-squared test and multivariate logistic regression analysis, age (≥50 years) is found associated with CDDP-induced nephrotoxicity, with odds ratio (OR) of 9.167 and 11.771, respectively. Three- and 18-month-old mice were employed to study the age-dependent susceptibility of CDDP-induced nephrotoxicity. Biochemical parameters, histopathogical examination, and mRNA biomarkers indicated that old mice were subjected to more severe kidney injury. In addition, old mice accumulated more CDDP in kidney than young mice, and the protein level of CDDP efflux transporter, MATE1, in aged mice kidney was 35 % of that in young mice. Moreover, inflammatory receptor TLR4 was higher in the kidney of old mice, indicating the alteration of inflammatory signaling in old mice. After CDDP administration, the induced alterations of TNF-α, ICAM-1, and TLR4 were more extensive in old mice. To summarize, aging increased the susceptibility of CDDP-induced renal function decline or nephrotoxicity.

**Electronic supplementary material:**

The online version of this article (doi:10.1007/s11357-015-9844-3) contains supplementary material, which is available to authorized users.

## Introduction

Cisplatin (CDDP) is a long used inorganic metal drug, with a wide usage in the treatment of solid tumors such as head and neck, lung, testis, ovarian, and bladder cancers. Although many other platinum antitumor agencies with reduced toxicity have been developed, CDDP is still favored in antitumor treatment given its defined therapeutic effect.

The most common side effects of cisplatin include ototoxicity, gastrointestinal and hematologic toxicities, acute kidney injury, and chronic kidney function decline (Evans et al. 1981; Loehrer and Einhorn 1984). Due to the high prevalence rate and severity of nephrotoxicity, the duration of CDDP chemotherapy is often terminated, affecting the prognosis. In the past decade, the investigation of CDDP-induced nephrotoxicity became the focus of acute kidney injury (AKI) study. The mechanism of cisplatin-induced nephrotoxicity is complicated, involving apoptosis, inflammation, mitochondria dysfunction, ROS, DNA damage, autophagy and the specific accumulation in S3 segment of renal tubules (Pabla and Dong 2008; Ozkok and Edelstein 2014). Recently, many studies have highlighted the role of CDDP accumulation in CDDP-induced nephrotoxicity. It was demonstrated that CDDP was transported into proximal tubular cells through the basal transporters OCT2 (Franke et al. 2010) and CTR1 (Pabla et al. 2009) from plasma, and excreted into urine via MRP2 (Wen et al. 2014) and MATE1 (Nakamura et al. 2010) located at the apical membrane. Recently, Masayuki et al. (Oda et al. 2014) reported that the rhythmic oscillations of renal OCT2 protein levels by circadian clock was a possible mechanism of dosing time-dependent changes in CDDP-induced nephrotoxicity. In addition, drug interaction on and the genetic variations of CDDP transporters were also found in a relationship to CDDP-induced nephrotoxicity (Tanihara et al. 2009; Iwata et al. 2012; Zhang and Zhou 2012; Sprowl et al. 2013).

A retrospective analysis from American head and neck cancer patients demonstrated that elder patients were more susceptible to CDDP-induced toxicities including renal toxicity (Argiris et al. 2004). In support of this, a recent study in Indonesia reported that age ≥50 years was correlated to CDDP-induced renal function decline (odd ratio = 3.4) (Prasaja et al. 2015). However, there are also studies against this positive relationship between age and CDDP nephrotoxicity (Cubillo et al. 2001; Moscetti et al. 2005). In the present study, we analyzed the data from Chinese lung cancer patients with CDDP-based chemotherapy and further compared the CDDP-induced nephrotoxicity in young mice with that in old mice.

## Materials and methods

### Chemicals

CDDP was purchased from Sigma-Aldrich (St. Louis, MO, USA) and dissolved in 0.9 % saline. MRP2 (rabbit polyclonal anit-mouse) and CTR1 (rabbit polyclonal anit-mouse) were purchased from Abcam (Cambridge, MA, USA), and OCT1, OCT2, TNF-α, and IL-1β were purchased from Proteintech (Wuhan, Hubei, China). MATE1 and Nrf2 were purchased from Santa Cruz Biotechnology (Dallas, TX, USA). ICAM-1 and TLR4 were purchased from Bioworld (St. Louis Park, MN, USA). All secondary antibodies were obtained from Santa Cruz Biotechnology.

### Patients

We retrospectively reviewed lung cancer patients who were admitted to Hunan Cancer Hospital, Central South University, from 2011 to 2015. Patients were treated with CDDP and given routine nephrotoxicity prevention procedure of hydration or treated with CBP. Creatinine clearance is an estimated glomerular filtration rate (eGFR) calculated by the Cockroft-Gault (CG) equation. Only the first chemotherapy cycle was evaluated. Patient’s nephrotoxicity was defined as renal function decline characterized by the change of eGFR to baseline lower than −30 %, which was the approximately the minimum value in patients with CBP-based therapy. Patients without the data of serum creatinine before CDDP infusion and after CDDP infusion were excluded. Moreover, patients who administrated other potential other nephrotoxic drugs (anti-HIVs, NSAIDs, vancomycin, aminoglycoside antibiotics) and pretreated with platinum-based chemotherapy were excluded. Variables studied as risk factors of renal function decline were age (<50 and ≥50 years old), gender, BMI, and comorbidities (hypertension and diabetes mellitus).

### Animal treatment

C57BL/6 mice (male, 3- and 18-month-old) were purchased from Hunan SJA Laboratory Animal Corporation and maintained in accordance with the guidelines for the care and use of laboratory animals as issued by the Center for Scientific Study with animal models of Central South University. The mice were acclimatized under normal environmental conditions and allowed free access to standard chow and tap water for 1 week before experimentation.

Mice of both age groups (18 per group) were randomized to receive either saline intraperitoneal injection or CDDP intraperitoneal injection (15 mg/kg). All mice were weighed before CDDP administration, and animals were euthanized 72 h post-CDDP injection, after which the abdominal cavity was opened for inspection of the kidneys. Part of the kidney was incised and fixed in formalin for histological examination, while the resulting parts were kept in liquid nitrogen for the extraction of messenger RNA (mRNA) and protein, as well as for CDDP quantification. At the same time, a blood sample was obtained by orbital puncture and analyzed for urea, creatinine, and other parameters. This study was approved by the Independent Ethics Committee Institute of Clinical Pharmacology, Central South University. Five-micron paraffin-embedded kidney sections were stained with H&E and were examined for histopathological changes by a board-certified pathologist from Xiangya Hospital. Neutrophils were counted from five to six mice per group in three nonoverlapping high-power fields (HPF). Immunofluorescence analysis was performed for Nrf2. Sections were washed and incubated with goat anti-rabbit IgG (Boster, Wuhan, China).

### Blood biochemistry

Blood creatinine (Cr), blood urea nitrogen (BUN), and aminotransferase (ALT) were determined on a fully automated clinical chemistry analyzer, LWC400 (Shenzhen, China).

### CDDP detection

Plamas and kidney samples from mice 4 to 72 h after injection were analyzed for CDDP concentration. The biosamples were weighed or quantified before dissolving in the 8 mL 70 % nitric acid and 2 mL 30 % hydrogen peroxide. The mix was then digested on electric heating panel for overnight and diluted to 5 mL. The resulting lysate was centrifuged at 12,000 rpm for 10 min, and 50 μL supernatant was diluted by adding 450 μL internal control solution of mass spectrometry analysis containing 10 ng/mL iridium and 0.1 % Triton X-100. The platinum content was measured by inductively coupled plasma mass spectrometry in the Analytical Center of Kemen Corporation (Changsha, Hunan, China).

### Reverse transcription and real-time qPCR

Total RNA was isolated from the renal cortex tissues using TRIzol and phenol-chloroform method. The total RNA (1 μg) was reversely transcribed using a high capability reverse transcription kit with DNA erase (Takara) and then diluted ten times. Five microliters was then used as the template for real-time PCR. The real-time qPCR was formed in duplicate using specific primers of the targeted genes (Supplementary Table 1; PrimerBank of Harvard Medical School), 5 μL cDNA templates, and 10 μL two-step SYBR green I mixture (Takara). The real-time qPCR condition was as follows: an initial 5-min preincubation step at 95 °C, followed by 40 cycles of two-step at 94 °C for 30 s and 60 °C for 60 s. Real-time PCR was performed on Roche 480. Relative changes in gene expression were calculated as fold changes using the comparative ΔΔCt method (where Ct is threshold cycle). Mice glyceraldehyde-3-phosphate dehydrogenase (GAPDH) housekeeper was used as the housekeeping gene for normalizing transcript levels.

### Western blotting

Renal tissue lysate was prepared by homogenation in lysis buffer (50 mM Tris, pH 7.5, 150 mM sodium chloride, 1 mM phenyl-methylsulfonyl fluoride, 1 mM sodium orthovanadate, 1 % Nonidet P-40, 50 mM sodium fluoride, 10 mg/mL proteinase inhibitors mixture, 10 % glycerol) at 4 °C, followed by the centrifugation at 14,000 rcf at 4 °C for 10 min. Protein concentration was measured by the Bradford assay, using bovine serum albumin as standard. Proteins (25 μg/lane) were separated by SDS-PAGE and then transferred to polyvinylidene difluoride membranes (Millipore, Billerica, MA). Nonspecific binding was blocked by preincubation of the PVDF membrane in Tris-buffered saline containing 0.1 % Tween 20 (TBS-T) and 5 % skimmed milk for 1 h. The PVDF was then incubated overnight at 4 °C with primary antibodies. After being washed, blots were then incubated with HRP-labeled secondary antibody and washed before bands were revealed using the BioRad imaging system. Band densities were determined using the β-actin or housekeeping proteins, and quantitation was determined using Image-Pro Plus software.

### Statistical analysis

Statistical analysis was conducted by Statistical Products Social Science (SPSS) 19 for Windows. Experimental values are expressed as mean ± SEM. Comparison of mean values between groups was performed by unpaired *t* test or one-way analysis of variance (one-way ANOVA) followed by post hoc Tukey test. *P* < 0.05, *P* < 0.01, and *P* < 0.001 were considered to be significant. Chi-squared test was used to analyze the relationship between risk factors (age, gender, BMI, and comorbidities) and nephrotoxicity incidence after the first chemotherapy cycle. Finally, multivariate analysis with logistic regression test was used to analyze the risk factors for nephrotoxicity in patients taking CDDP.

## Results

### Clinical observation

**Fig. 1:**
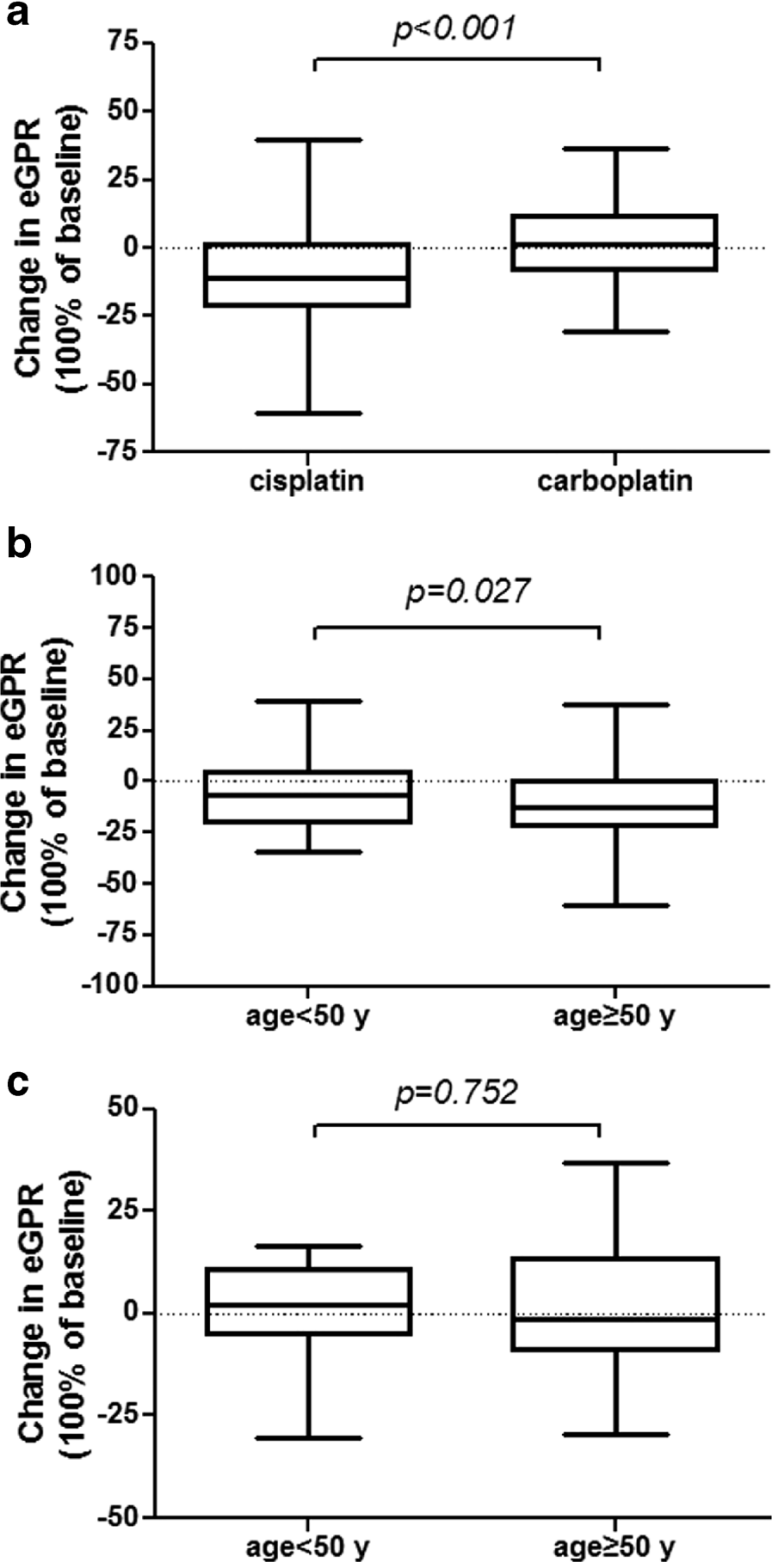
Change of eGFR in the patients with CDDP- and CBP-based therapies. **a** Change of eGFR (100 % of baseline) in the patients (*n* = 182) treated with CDDP after the first cycle of chemotherapy was significantly below that in carboplatin-treated patients (*n* = 58). **b** Among the patients with CDDP-based therapy, change of eGFR decreased significantly in patients with the age ≥50 than <50 years. **c** In patients with CBP-based therapy, there was no change in the alteration of eGFR between the age ≥50 and <50 years

In this study, a total of 240 subjects of lung cancer patients were recruited in accordance with the criteria of subject recruitment. Patients who received CDDP treatment were 182 people, while the patients with CBP chemotherapy were 58. Table 1 describes the general and clinical characteristics of these subjects. Three to four days after platinum dosing, eGFR decreased significantly in patients receiving CDDP injection compared to CBP injection (Fig. 1a). With respective to the change of eGFR, it decreased significantly in CDDP-treated patients with the age ≥50 than <50 years (Fig. 1b), whereas the change of eGFR was similar between the two age groups of CBP-treated population (Fig. 1c). Table 2 shows the factors affecting CDDP-induced decline of renal function by using chi-squared test. Among these factors, only age (*p* = 0.011, OR = 9.167, 95 % CI = 1.192–70.475) had a significant relationship with renal function decline after the first cycle of CDDP chemotherapy. Multivariate analysis using logistic regression showed that age (*p* = 0.020, OR = 11.771, 95 % CI = 1.470–94.266) had a significant association with the decline of renal function, while other factors still had no statistically significant relationship with renal function decline.

**Table 1:** Patient clinicopathologic characteristics and chemotherapy regimens

	Cisplatin	Carboplatin
Patients number	182	52
Age (mean ± SD, years)	54.18 ± 0.63	56.52 ± 1.27
Sex (female/male)	33/148	19/32
BMI (≥25)	31	7
Hypertension	13	6
Diabetes	6	2
Chemotherapy regimens		
Gemcitabine	83	4
Paclitaxel	30	27
Docetaxel	23	5
Pemetrexed	32	15
Etoposide	5	3
Others	9	4

**Table 2:** Factors affecting renal function decline induced by CDDP

	ΔeGFR < −30 %		ΔeGFR > −30 %		*P* value	OR (95 % CI)
	*n*	%	*n*	%		
Gender						
Female	5	15.2	28	84.8	0.328	1.722 (0.574, 5.169)
Male	14	9.4	135	90.6		
Age						
≥50	18	14.3	108	85.7	0.011	9.167 (1.192, 70.475)
<50	1	1.8	55	98.2		
BMI						
≥25	1	3.2	30	96.8	0.149	0.246 (0.032, 1.918)
<25	18	11.9	133	88.1		
Diabetes						
Yes	1	16.7	5	83.3	0.612	1.756 (0.194, 15.871)
No	18	10.2	158	89.8		
Hypertension						
Yes	2	16.7	10	83.3	0.465	1.800 (0.364, 8.904)
No	17	10	153	90		

### Body weight change and biochemical parameters

During the experiment, two mice in the aged group died 2 days after the CDDP injection. CDDP decreased body weight by 15 and 16 % in aged and young groups, respectively. There was no remarkable change in body weight of control mice at either age (Table 3). Results of blood biochemistry for nephrotoxicity and hepatotoxicity are shown in Table 3. CDDP treatment significantly increased the levels of BUN, Cr, and ALT in old aged group, but not in young group.

**Table 3:** Change of body weight and serum plasma biochemical indicators after CDDP injection in mice (*n* = 7–9)

Age	Treatment	BW change (%)	BUN (mmol/L)	Cr (μmol/L)	ALT (U/L)
3-month old	NS	−0.2 ± 0.83	9.94 ± 0.74	36.1 ± 2.54	32.8 ± 2.57
	CDDP	−16.56 ± 0.44*	18.64 ± 2.94	44.55 ± 3.01	37 ± 3.85
18-month old	NS	−0.16 ± 0.53	15.03 ± 1.73	53.73 ± 3.18	42.5 ± 3.99
	CDDP	−15.8 ± 1.49*	47.16 ± 2.91*^,^**	168.0 ± 36.1*^,^**	68.3 ± 6.96*^,^**

*P < 0.05, compared with NS control; **P < 0.05, compared with 3-month-old group of CDDP treatment

### Histopathogical examination

**Fig. 2:**
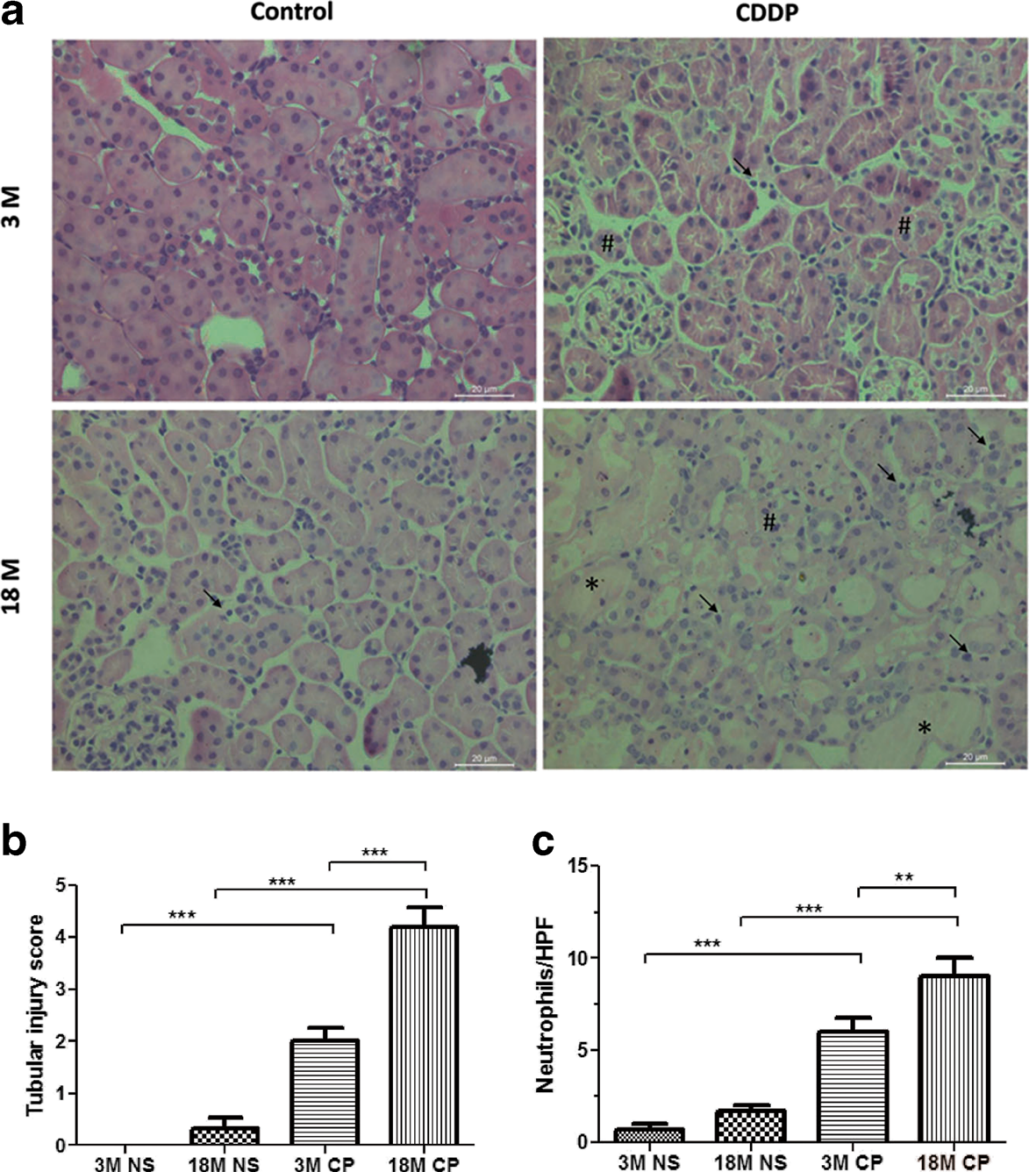
Histopathologic injury in young and old mice treated with CDDP. Three- and 18-month-old mice were treated with vehicle or 15 mg/kg of i.p. CDDP, and kidneys were collected 72 h after CDDP injection. Samples were fixed in formalin before routine processing and paraffin embedding. **a** Five-micron sections of kidneys were stained with hematoxylin and eosin and were examined by light microscopy for the presence and severity of renal cast formation (*asterisk*) and proximal tubule degeneration (*number sign*) (×400 magnification). **b** Histological damage grades were scored by two pathologists based on the percentage of tubule degeneration and necrosis (0 = no injury, 1 = <10 %, 2 = 10–25 %, 3 = 26–40 %, 4 = 40–50 %, 5 = >50 %). **c** The number of neutrophils were quantified in hematoxylin-and-eosin-stained kidney sections from vehicle or CDDP-treated mice. *n* = 5–6. *P < 0.05, **P < 0.01, ***P < 0.001

There was no apparent renal histopathology change in control mice of both ages. However, following treatment with CDDP, remarkable proximal tubule cell necrosis, proximal tubule degeneration, and hyaline casts were observed (Fig. 2a, b). Although the glomerulus did not appear to be affected by CDDP treatment in both age groups, the proximal tubule damages were more extensive in old age group compared with the young. In addition, more neutrophil infiltration was found in old mice kidney than the young after CDDP injection (Fig. 2c).

### mRNA biomarkers of CDDP-induced nephrotoxicity

**Fig. 3:**
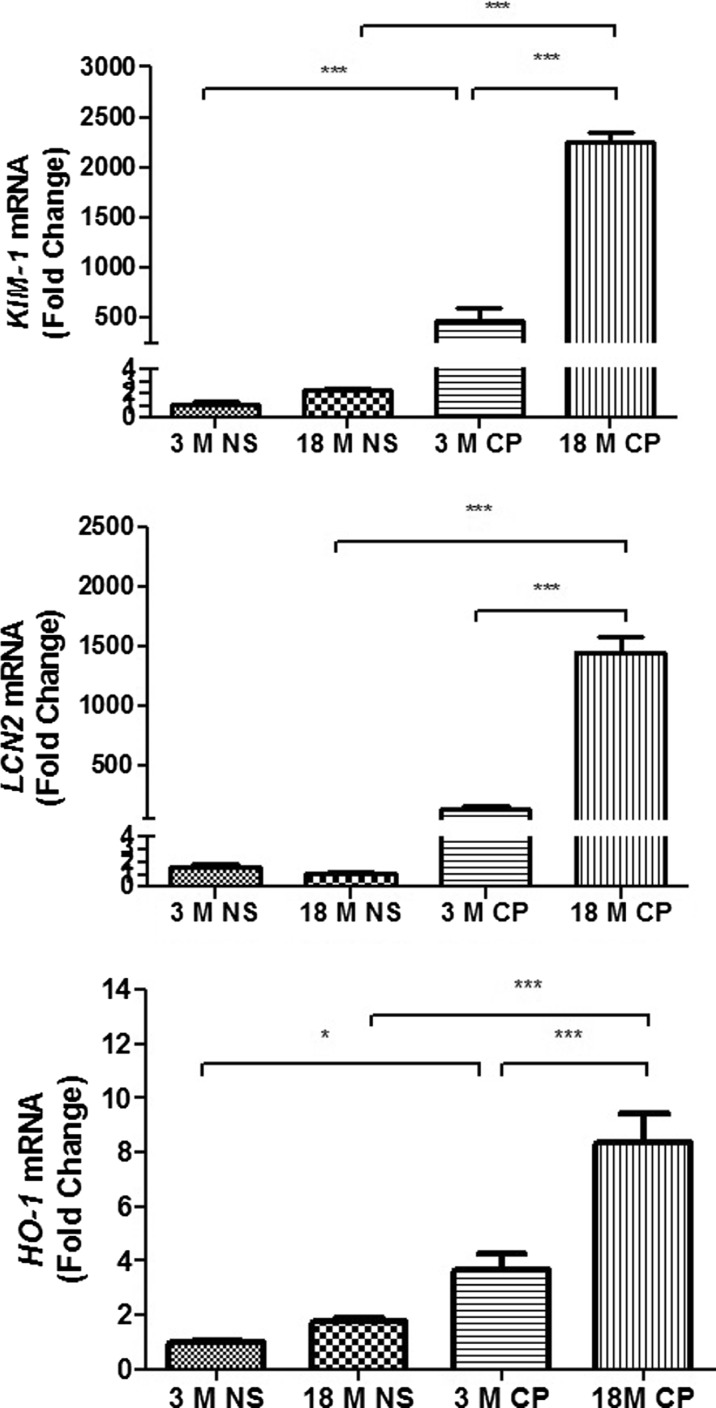
mRNA biomarkers of CDDP-induced nephrotoxicity. The mRNA levels of Kim1, Lcn2, and HO-1 were measured by RT-qPCR. *n* = 6 to 9. *P < 0.05, **P < 0.01, ***P < 0.001

mRNA molecules related to kidney injury were examined in the mice receiving CDDP in both age groups. The transcript levels of the Kim-1, Lcn2, and HO-1, which are the indicators of AKI, were remarkably elevated by CDDP treatment (Fig. 3).

### Antioxidant pathway related to CDDP-induced nephrotoxicity

**Fig. 4:**
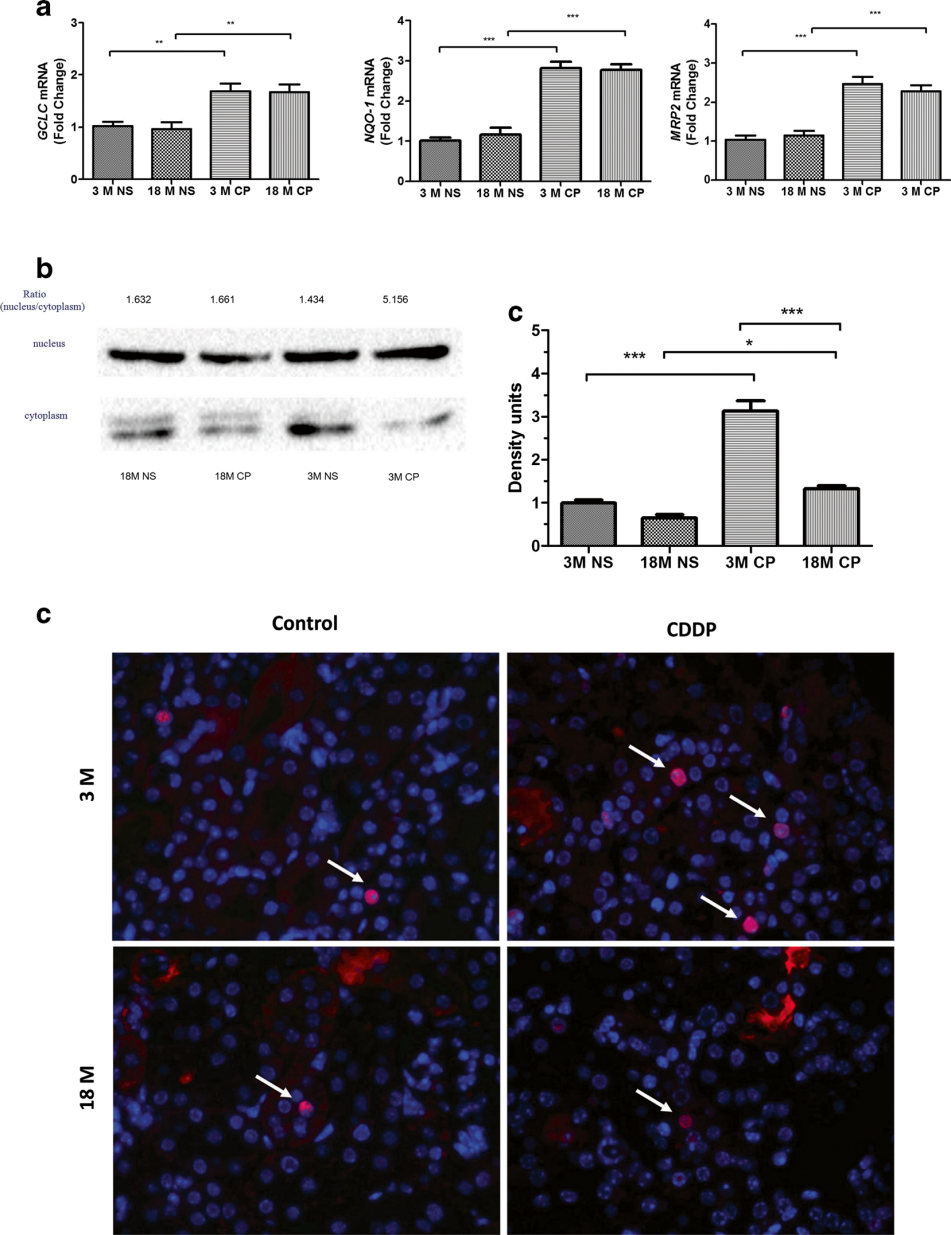
The change of antioxidant pathway related to CDDP-induced nephrotoxicity. **a** The mRNA levels of GCLC, NQO-1, and MRP2 in mice kidney from vehicle or CDDP-treated mice (*n* = 6 to 9). **b** The protein level of Nrf2 in the nucleus and cytoplasm from renal kidney cells. **c** Immunofluorescent staining of Nrf2 in kidney sections of young and old mice treated with cisplatin (*blue*, DAPI staining; *red*, Nrf2; *white arrow*, nucleus staining of Nrf2). *P < 0.05, **P < 0.01, ***P < 0.001

The transcriptional expression of antioxidation genes HO-1, GCLC, and NQO-1 that were overexpressed by CDDP treatment can scavenge the excessive reactive oxidative species induced by CDDP. It was also reported that HO-1, GCLC, and NQO-1 were regulated by Nrf2/Keap1 signals and can be the indicators of CDDP-induced nephrotoxicity (Aleksunes et al. 2010). The detoxification products of CDDP can be extruded via MRP2 which was upregulated by Nrf2. Thus, we further detected the mRNA levels of these molecules (Figs. 3 and 4a). All these genes were transcriptionally upregulated by CDDP treatment. However, only CDDP-induced upregulation of HO-1 mRNA was more profound in old mice kidney than that in young mice. There was no difference on the increasing of GCLC, NQO-1, and MRP2 by CDDP treatment in both age groups. Furthermore, Western blot and immunofluorescence (IF) showed that the nuclear translocations of Nrf2 in old mice were more than that in young mice (Fig. 4).

### CDDP concentration

**Fig. 5:**
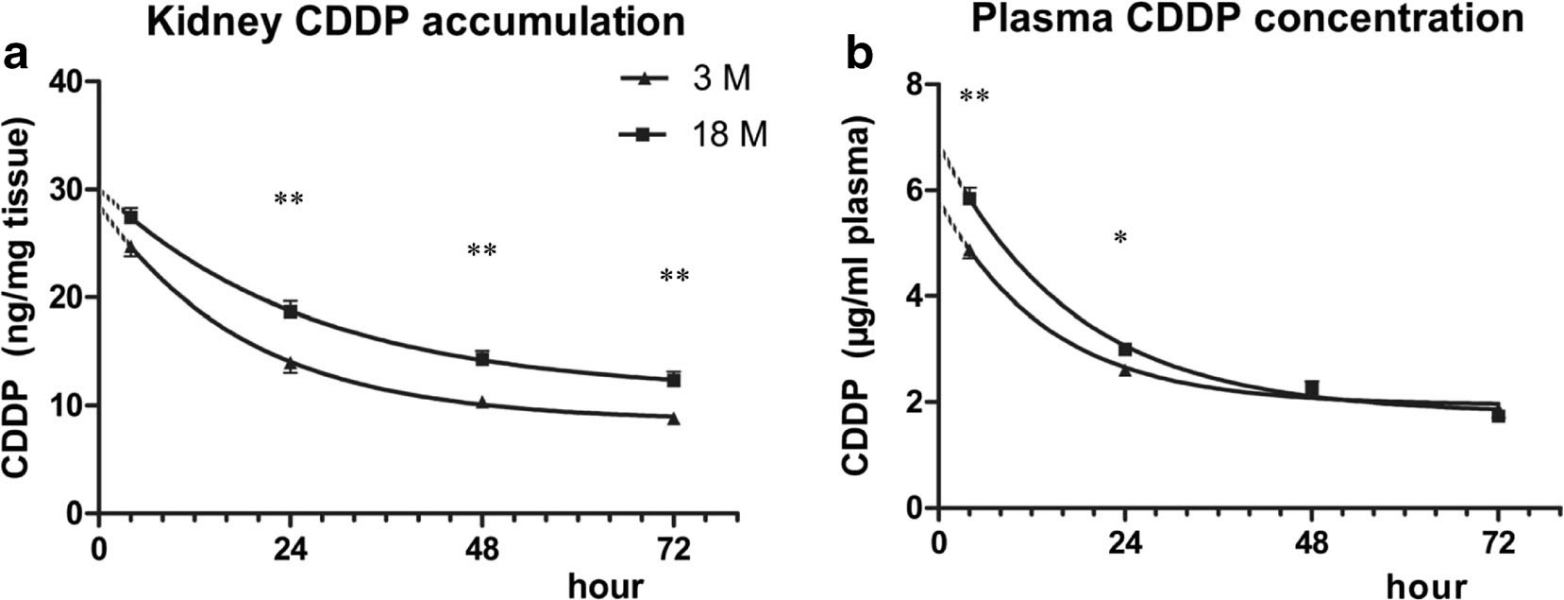
CDDP concentrations in the kidney of 3- or 18-month-old mice treated with CDDP. The concentrations were quantified by inductively coupled plasma mass spectrometry in the kidneys (**a**) and plasma (**b**) of young and old mice treated with 15 mg/kg of i.p. cisplatin. *n* = 4 to 6. *P < 0.05, **P < 0.01

Renal specific accumulation of CDDP is tightly related to the severity of its nephrotoxicity. Using ICP-MS detection method, it showed that renal concentrations of CDDP in old mice were significantly higher than that in young mice at the time of 24, 48, and 72 h post-CDDP injection (Fig. 5a). Additionally, the old mice had a 30 % increase in the area under the curve over the young group. At 4 and 24 h post-CDDP injection, the plasma concentrations of CDDP were higher in old group than those in young group (Fig. 5b), indicating a lower plasma CDDP clearance of old mice. However, the plasma concentrations were comparable between the two groups at the time of 48 and 72 h post-CDDP injection.

### Kidney uptake and efflux transporters

**Fig. 6:**
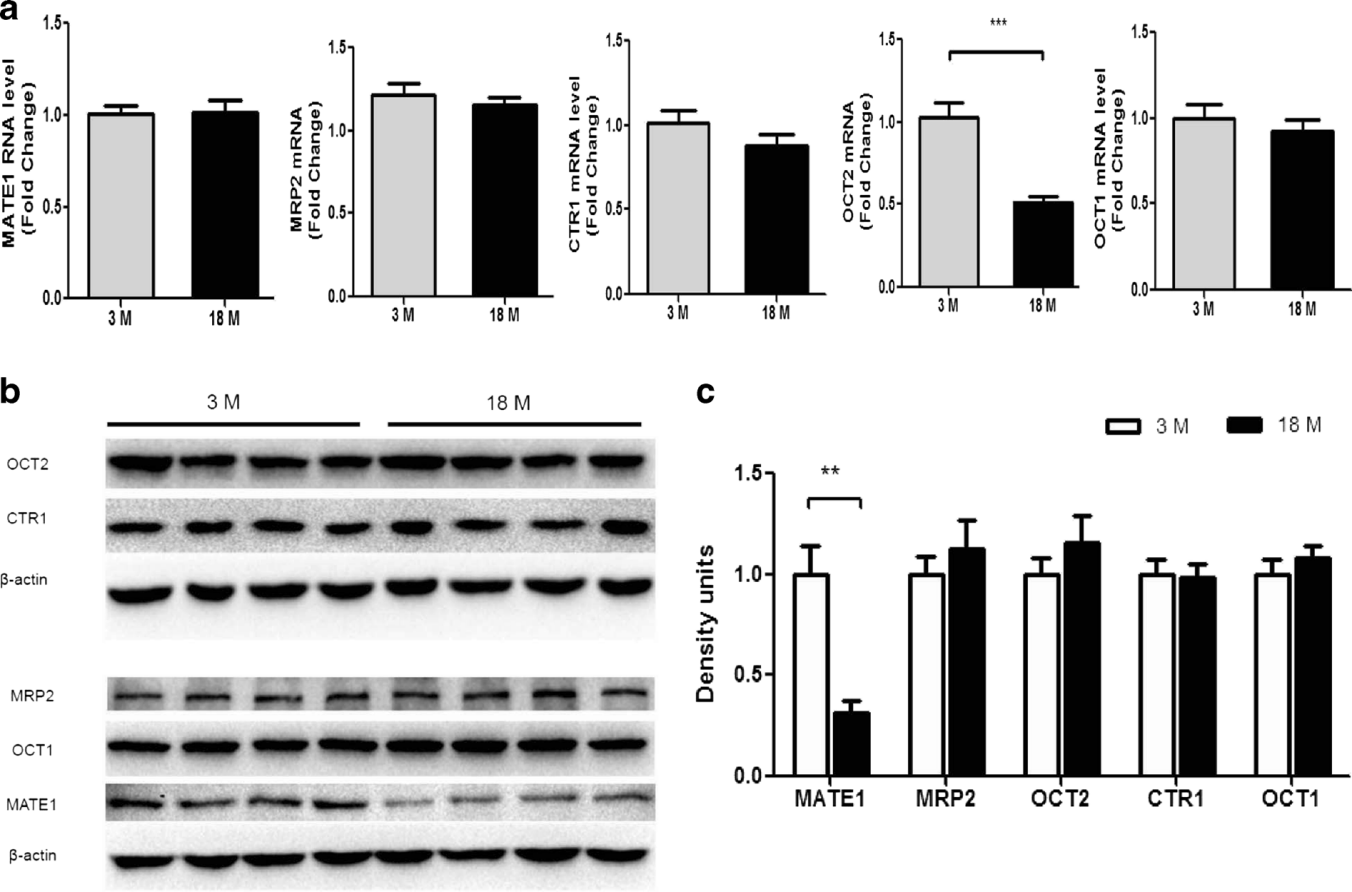
Change of cisplatin transporters in the kidney of 3- or 18-month-old mice. **a** The mRNA levels of OCT1, OCT2, CTR1, MATE1, and MRP2 were measured (*n* = 7 to 9). **b** Expression levels of OCT1, OCT2, CTR1, MATE1, and MRP2 were measured by Western blot analysis using their respective antibodies. **c** The relative ratio of OCT1, OCT2, CTR1, MATE1, and MRP2 to β-actin was determined by densitometry and are reported as mean ± SEM (*n* = 4). *P < 0.05, **P < 0.01, ***P < 0.001

Given the fact that old mice accumulated more CDDP in their kidneys, it was easily to conceive that the age-dependent change of transporters might have an influence on renal tubule-mediated uptake and excretion of CDDP. Thus, we quantified both the mRNA and protein levels of CDDP uptake transporters OCT1, OCT2, and CTR1, as well as the efflux transporters, MATE1 and MRP2 (Fig. 6). Among these transporters, only OCT2 mRNA levels were significantly downregulated in old mice kidney, whereas its protein levels kept unchanged. There was nearly 2-fold decrease in MATE1 protein levels in old mice kidney compared with young mice kidney, whereas the renal MATE1 mRNA levels of the two age groups were basically the same.

### Changes of inflammation related to CDDP-related kidney injury

**Fig. 7:**
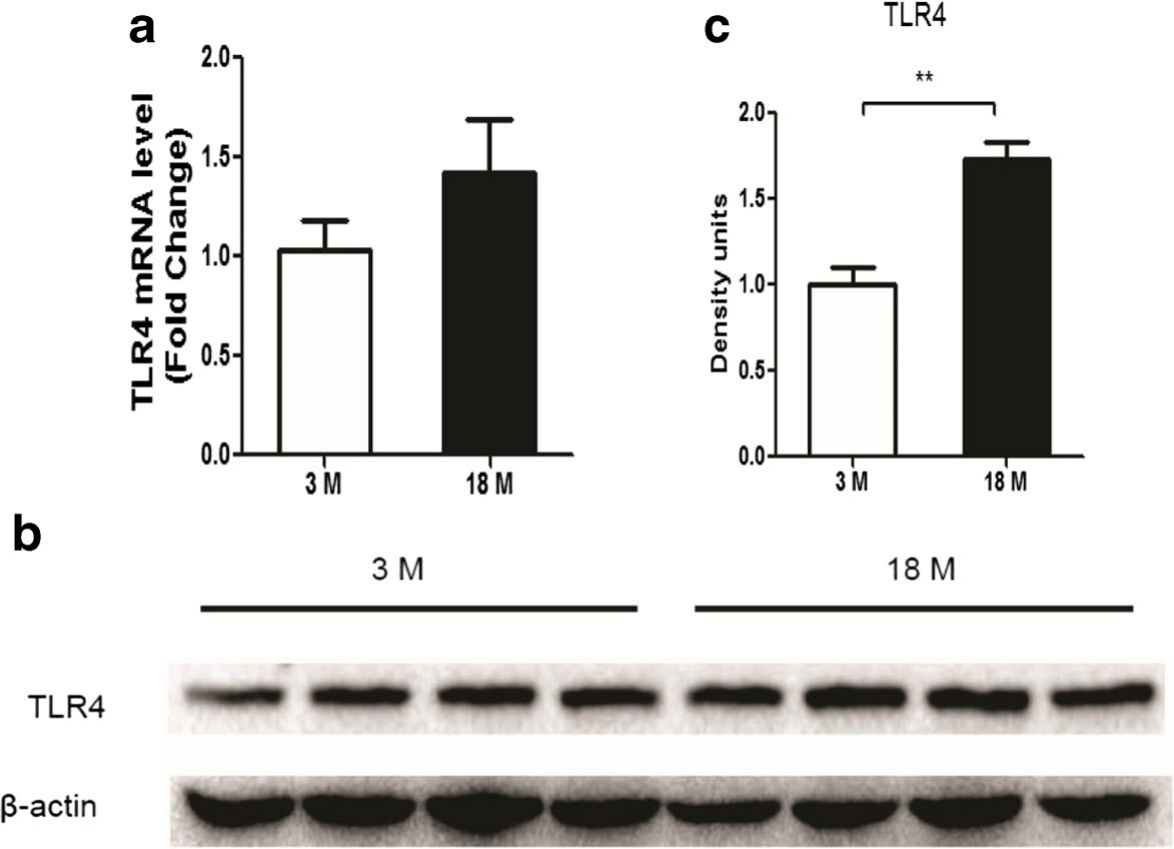
Expression levels of TLR4 in the kidney of young and old mice. The mRNA levels (**a**) (*n* = 5–8) and protein levels (**b**) of TLR4 were measured. **c** Densitometric analysis of TLR4 to β-actin ratio on Western blot (*n* = 4). **P < 0.01

**Fig. 8:**
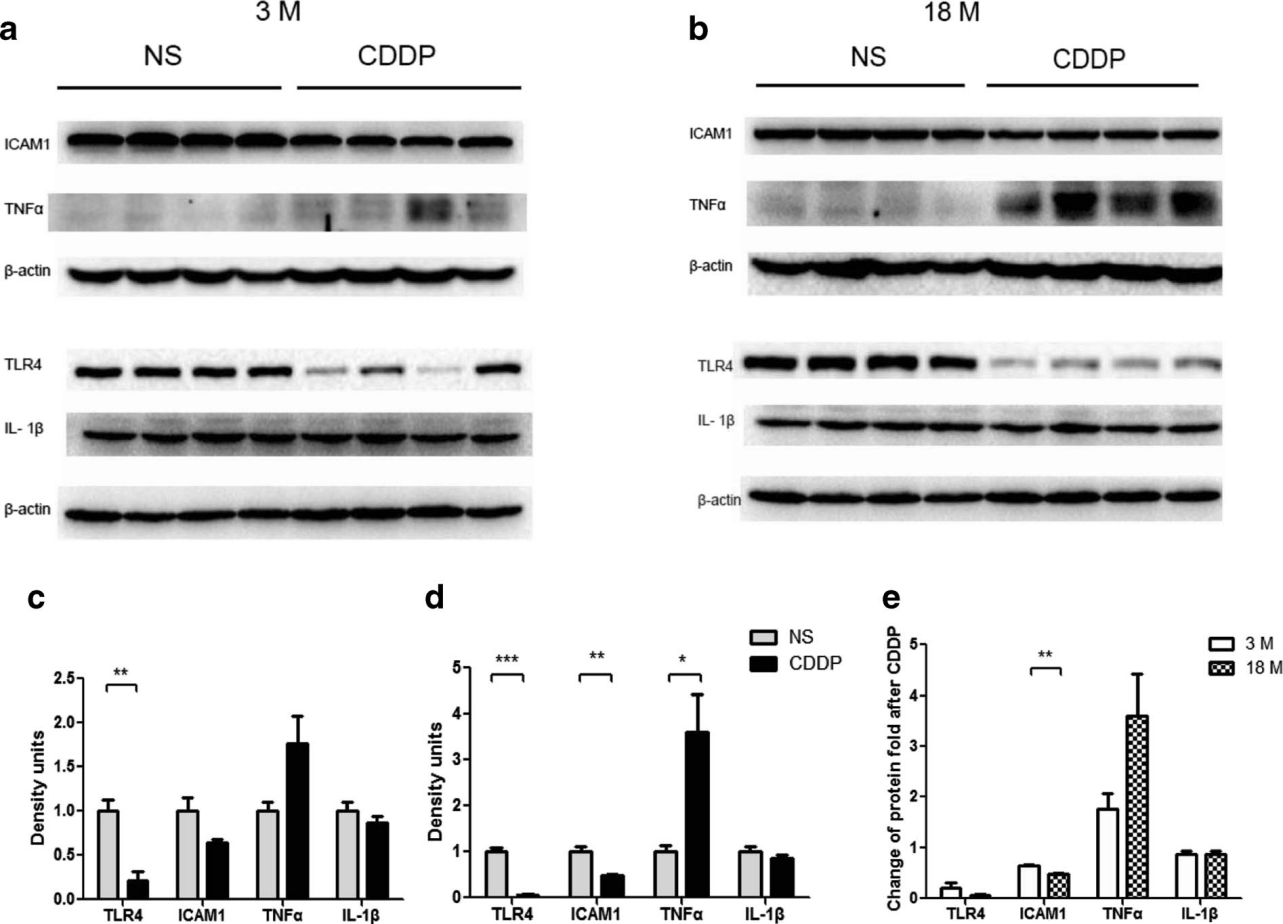
Change of inflammatory factors in the kidney of 3- or 18-month-old mice treated with cisplatin. Protein expression levels of ICAM-1, TNF-α, TLR4, and IL-1β in young (**a**) and old (**b**) mice treated with NS and CDDP were measured. Densitometric analysis of the ratio of ICAM-1, TNF-α, TLR4, and IL-1β to β-actin in young (**c**) and old (**d**) mice groups are shown. **e** The change ratios of ICAM-1, TNF-α, TLR4, and IL-1β protein in CDDP-treated mice to control mice in old mice group were compared with that in young group. *n* = 4. *P < 0.05, **P < 0.01, ***P < 0.001

Inflammatory response is an important mechanism leading to CDDP-related kidney injury. TLR4, the most thoroughly studied member of the TLR family, can initialize the process of inflammation (e.g., the upregulation of TNF-α). Figure 7 shows that the protein levels of TLR4 were significantly increased in the kidneys of old mice, while there was a nonsignificant increasing trend in its mRNA levels. After CDDP injection, TNF-α levels were increased, TLR4 and ICAM-1 were decreased, and IL-1β was not changed in both age groups (Fig. 8). Moreover, CDDP-induced decline of TLR4 and ICAM-1 and augment of TNF-α were more profound in the old group than in the young (Fig. 8). Interestingly, all the renal mRNA levels of TLR4, ICAM-1, TNF-α, and IL-1β were augmented after CDDP administration, even with a higher increasing trend in old age group than young group (data are shown in Supplementary Fig. 1).

## Discussion

In the present study, we investigated the association between nongenetic factors and CDDP-induced renal clearance decline, and the difference of CDDP-induced nephrotoxicity in the two age groups of C57BL/6J mice. By the retrospective analysis of the renal function data from the patients, we found that the old age was an independent risk factor to the decline of renal function. In the mice model with the two age groups, CDDP-induced nephrotoxicity was also significantly more severe in the old mice than the young mice.

Although the BUN and Cr of the young mice after CDDP injection did not significantly increase, there were some abnormalities in histological examination of the renal proximal tubules. In the guideline of cancer therapy, CTCAE4.0, a 1.5-fold increase of serum Cr is considerate as grade 1 AKI. However, it is commonly regarded that Cr will have an obvious increase only when the nephrons are largely damaged. Recent studies of renal toxins revealed that urine Kim1, NGAL, and other molecules outperform the traditional biomarkers of nephrotoxicity (Mishra et al. 2004; Gautier et al. 2010; Vizeacoumar et al. 2010). In our previous study (Li et al. 2013), the mRNA levels of Kim1 and Lcn2 were remarkably increased, despite that the BUN and Cr did not have the equivalent elevating trends. From the data of 182 patients with CDDP chemotherapy, only one patient had the 1.5-fold increase in serum Cr after CDDP injection. Nonetheless, most of these patients have a slighter Cr increase than the patients with CBP chemotherapy. Although urine Kim1 and NGAL were the highly approved biomarkers for the indication of nephrotoxicity in animal trials, they are currently not applied clinically. It is, therefore, extremely pivotal to validate these renal biomarkers in human clinical trials.

In the association study of the nongenetic factors and CDDP nephrotoxicity, the selection of the endpoint is of great importance. In a recent study of Indonesian cancer patients, Yenny et al. (Prasaja et al. 2015) chose renal function decline characterized by creatinine clearance <60 ml/min as the nephrotoxicity criterion. It is a kind of abitrary because the creatinine clearance after CDDP injection is also tightly related to the baseline value of creatinine clearance, which was not adjusted in this study. According to other studies (Tzvetkov et al. 2011; Sprowl et al. 2012), we chose the change ratio of eGFR as the indicator of renal function. To determinate the boundary of nephrotoxicity, we abitrarily employed the change of eGFR to baseline lower than −30 %, which seldom happened in patients with CBP-based chemotherapy. To eliminate the inference from the multiple cycles of CDDP chemotherapy, only the data of the first CDDP chemotherapy was included. The finding that old age was a risk factor of CDDP nephrotoxicity was further supported by the experiments in aged mice.

In a study from The Jackson Laboratory, the old age of mice was regarded as a period when senescent changes can be detected in almost all biomarkers in all animals (The Jackson Laboratory 2011). For C57BL/6J mice, the lower limit of old is 18 months, whereas 3 months old was thought to be the mature adult. Thus, in the design of experiment, we chose 3- and 18-month-old mice as the young and old age groups, respectively. After CDDP injection (15 mg/kg), both the plasma BUN and Cr concentration in the old group had a 2-fold increase of that in control group, indicating a severe nephrotoxicity. Additionally, the mRNA biomarkers of kidney injury (Kim1, Lcn2, HO-1) and the results of histological analysis all supported that aged mice was more susceptible to CDDP-induced nephrotoxicity. Besides, two mice in old mice group died in the third day after CDDP injection, which could be caused by the severe nephrotoxicity.

There are two kinds of CDDP in plasma, the bound and unbound CDDP. Unbound CDDP is largely eliminated through renal filtration within 4 h post-injection (Vermorken et al. 1984; Vermorken et al. 1986). Moreover, CDDP is specifically accumulated in kidney, through the uptake transporters OCT1, OCT2, and CTR1, and excreted into urine via MATE1 and MRP2 at the apical membrane of proximal tubular cells. Our study demonstrated a higher CDDP accumulation in the kidney of old mice, in accordance with their severe nephrotoxicity. The 65 % decrease of MATE1 in old mice kidney can limit the extrusion of CDDP, causing more CDDP renal accumulation and severe nephrotoxicity. We also found that the mRNA of OCT2, but not the protein level, was downregulated in aged mice. However, in a study of aged rats, both the mRNA and protein levels were found decreased in old age group (Ren et al. 2014). It would be interesting to investigate the mechanism concerning the change of these transporters among different species. Besides, it was reported that the glomerular filtration and renal blood flow rates declined in a linear fashion after the age of 30 (Anderson and Brenner 1986; Muhlberg and Platt 1999). Therefore, the renal elimination of CDDP decreased in old mice, which resulted in high plasma CDDP concentration especially from 4 to 24 h after CDDP dosing. In this regard, the high plasma CDDP concentration could force more drugs into the kidney tubular cells via the basal membrane. In summary, both the downregulation of MATE1 and the decrease of CDDP filtration can contribute to the increased renal accumulation of CDDP.

The signaling of Toll-like receptors (TLRs) are widely expressed in leukocytes and kidney epithelial cells, regulating innate and adaptive immune responses, which is an important pathway leading to AKI (Anders et al. 2004). With respect to CDDP-induced nephrotoxicity, the knockout of TLR4 significantly decreased CDDP-induced inflammatory cytokines and its severity of nephrotoxicity (Zhang et al. 2008). In our study, we found that old mice expressed more TLR4 in kidney and also had a higher increase of TNF-α after CDDP dosing. Although higher CDDP accumulation in the kidney of old mice can produce more TNF-α, it cannot overlook the influence of age on inflammatory response. Mounting data have evidenced the age-related alterations in the inflammatory response to dermal injury (Swift et al. 2001), pneumonia (van Vught et al. 2014), brain trauma (Timaru-Kast et al. 2012), fibrosis (Mahrouf-Yorgov et al. 2011; Wolf et al. 2012), acute coronary syndrome (Badran et al. 2009), etc. Therefore, the upregulation of TLR4 that initiate the inflammatory response is partially responsible for the susceptibility of CDDP-induced nephrotoxicity in old mice. It was interesting that both the TLR4 and ICAM-1 were downregulated in renal tissues, but not IL-1β. It has been demonstrated that renal TLR4 protein shed into the urine after the stimulation of toxins, whereas the mRNA level were still increasing (Zager et al. 2007). Our results were in support of this finding. Moreover, we showed that the extent of TLR4 decreasing is related to the severity of CDDP nephrotoxicity. However, why this shedding happens and what is the outcome of TLR4 shedding are still not fully understood. It is also unclear whether the decrease of ICAM-1 and the unchanged IL-1β levels are caused by their secretion into plasma or shedding into urine.

Nrf2 is a cytoprotective factor in various pathological processes including CDDP-induced AKI. The expression of HO-1, Gclc, NQO-1, and MRP2, via activation of Nrf2, has the capacity to quench free radicals and electrophiles caused by CDDP. In addition, the levels of these molecules are thought to parallel to the severity of CDDP nephrotoxicity. Several studies have reported that aging is associated with diminished antioxidant capacity and increased accumulation of reactive oxygen and nitrogen species. Recently, Sellamuthu et al. (Gounder et al. 2012) demonstrated that the inducible antioxidant pathways of Nrf2 were impaired in the aging heart of mice, as evidenced by the decreased nuclear translocation of Nrf2, ARE-binding activity of nuclear Nrf2, and scavenging of reactive oxygen species. In our study, the levels of GCLC, NQO-1, and MRP2 were comparable in both age kidneys, whereas the CDDP accumulation and the nephrotoxicity severity were higher in old group. Thus, it is conceived that in aging kidneys, the antioxidant pathways of Nrf2 is also impaired, causing the relative lower level of GCLC, NQO-1, and MRP2. Western blot and IF were in support that age weakened the signaling of Nrf2 in mice kidney. Inconsistently, the induced levels of HO-1 were higher in aged mice than those in young mice. It is further important to reveal the protein levels of these antioxidant molecules induced by Nrf2 and the mechanism why age blunts Nrf2 activity.

Also, these are some other shortages in our study. In the animal experiment, the urine samples are not collected. Thus, we did not analyze the urinary nephrotoxicity biomarkers and the urine CDDP clearance, which can make the study more convincing. Moreover, only the retrospective clinical data was obtained and therefore, the gene polymorphisms of the patients are not available. It is our future work to investigate the factors of age and gene polymorphisms on the pharmacokinetics of CDDP in patients.

## Conclusion

The finding from this study demonstrated that old age was a risk factor to CDDP-induced renal clearance decline or nephrotoxicity. The results from the animal experiments showed that old mice accumulated more CDDP in kidney than young mice, which was highly related to the age-dependent susceptibility to CDDP-induced nephrotoxicity. Moreover, the altered inflammatory response and antioxidant signaling in aged kidneys partially contributed to the age-dependant susceptibility of the renal injury.

## Electronic supplementary material

Below is the link to the electronic supplementary material.

Supplementary Figure 1 The mRNA change of inflammatory signalings by CDDP treatment (JPEG 879 kb)

Supplementary Table 1 (DOC 38 kb)
