# Supplementary figures and images for: Aging increases the susceptibility of cisplatin-induced nephrotoxicity
Source: Age (Dordr). 2015 Nov 3;37(6):112. doi: 10.1007/s11357-015-9844-3 (PMC5005850; doi:10.1007/s11357-015-9844-3)

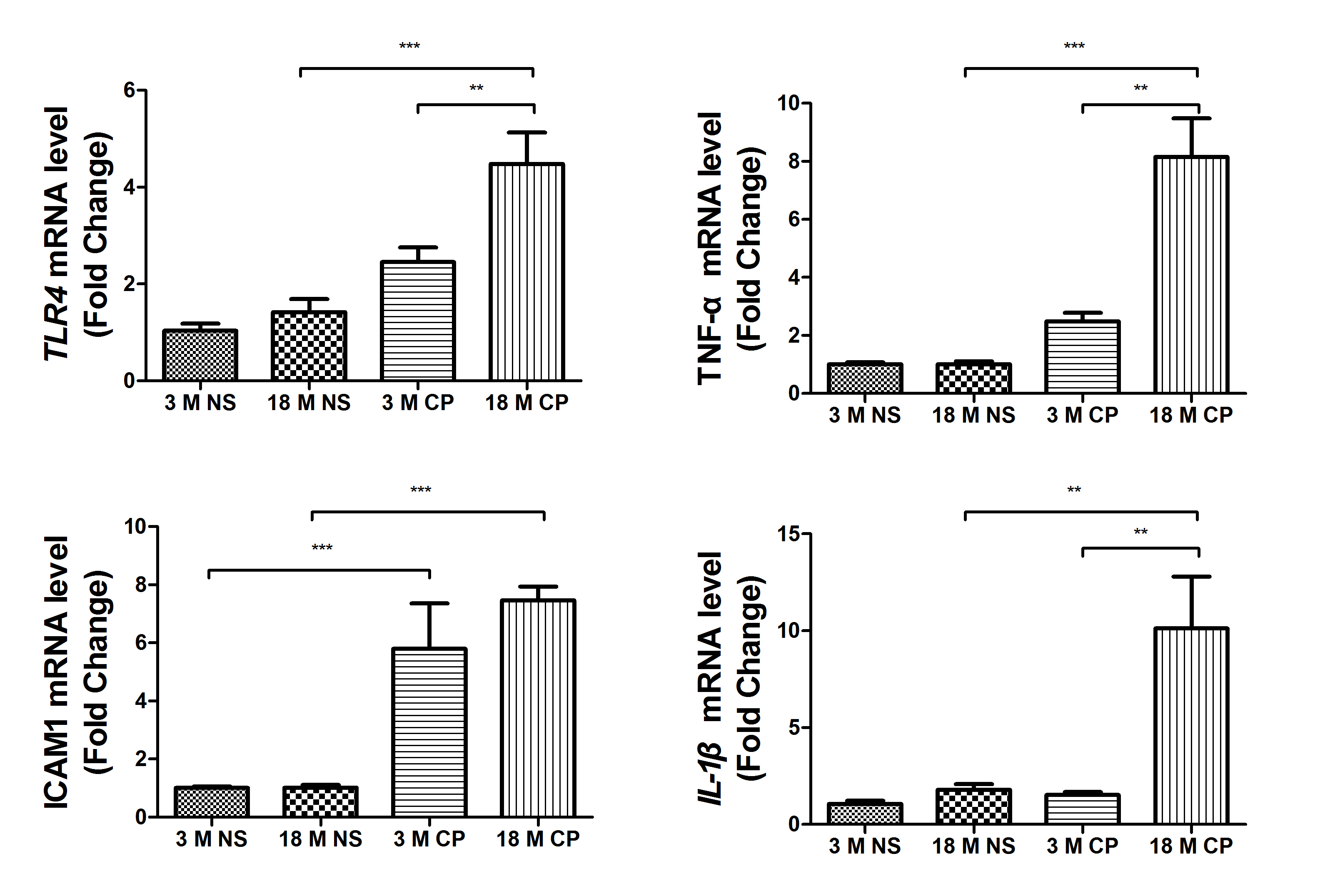

Supplement: Supplementary file 1 — The mRNA change of inflammatory signalings by CDDP treatment (JPEG 879 kb) [file 11357_2015_9844_Fig9_ESM.jpg]
