# Supplementary material for: Aging increases the susceptibility of cisplatin-induced nephrotoxicity
Source: Age (Dordr). 2015 Nov 3;37(6):112. doi: 10.1007/s11357-015-9844-3 (PMC5005850; doi:10.1007/s11357-015-9844-3)
Supplement: Supplementary file 2 — (DOC 38 kb) [file 11357_2015_9844_MOESM1_ESM.doc]

|  | Forward Sequence | Reverse Sequence |
| --- | --- | --- |
| CTR1 | 5'-GCCTTCGTGGCAGTGTTTTTA-3' | 5'-GCGAATGCTGACTTGAGACTTTC-3' |
| GAPDH | 5'- AGGTCGGTGTGAACGGATTTG-3' | 5'-GGGGTCGTTGATGGCAACA-3' |
| GCLC | 5'-ACACCTGGATGATGCCAACGAG-3' | 5'-CCTCCATTGGTCGGAACTCTAC-3' |
| HO1 | 5'-CACTCTGGAGATGACACCTGAG-3' | 5'-GTGTTCCTCTGTCAGCATCACC-3' |
| ICAM1 | 5'-GTGATGCTCAGGTATCCATCCA-3' | 5'-CACAGTTCTCAAAGCACAGCG-3' |
| IL-1β | 5'-GAAATGCCACCTTTTGACAGTG-3' | 5'-TGGATGCTCTCATCAGGACAG-3' |
| KIM-1 | 5'-AGCAGTCGGTACAACTTAAAGG-3' | 5'-ACTCGACAACAATACAGACCAC-3' |
| LCN2 | 5'-GGGAAATATGCACAGGTATCCTC-3' | 5'-CATGGCGAACTGGTTGTAGTC-3' |
| MATE1 | 5'-GTTGGCCTTACGGAGAGGAC-3' | 5'-AATCCCACCCACCAAGACTAA-3' |
| MRP2 | 5'-AGCAGGTGTTCGTTGTGTGT-3' | 5'-CAGGAGGAATTGTGGCTTGTC-3' |
| NOQ1 | 5'-GCCGAACACAAGAAGCTGGAAG-3' | 5'-GGCAAATCCTGCTACGAGCACT-3' |
| OCT1 | 5'-CAGGTTTGGCCGTAAGCTCT-3' | 5'-GCAACATGGATGTATAGTCTGGG-3' |
| OCT2 | 5'-TGGCAGGACATTTTGTAGGTG-3' | 5'-CCTGTCAGGAAGCGAAGTAAG-3' |
| TLR4 | 5'-GCCTTTCAGGGAATTAAGCTCC-3' | 5'-GATCAACCGATGGACGTGTAAA-3' |
| TNFα | 5'-GGTGCCTATGTCTCAGCCTCTT-3' | 5'-GCCATAGAACTGATGAGAGGGAG-3' |

Supplementary Table 1. Mouse real-time qPCR primers
